# Can chest CT improve sensitivity of COVID-19 diagnosis in comparison to PCR? A meta-analysis study

**DOI:** 10.1186/s43163-020-00039-9

**Published:** 2020-11-09

**Authors:** Heba Mahmoud, Mohamed Shehata Taha, Anas Askoura, Mohammed Aleem, Azza Omran, Soha Aboelela

**Affiliations:** 1 Ear, Nose and Throat Department, Faculty of Medicine, Ain Shams University, Cairo, Egypt; 2 Clinical Pathology Department, Al-Mataria Teaching Hospital, Cairo, Egypt; 3 Clinical Pathology Department, Faculty of Medicine, Ain Shams University, Cairo, Egypt

**Keywords:** SARS CoV-2, COVID-19, PCR, CHEST CT scan

## Abstract

**Abstract:**

**Background:**

In December 2019, SARS-CoV-2 was identified as the causative agent of pneumonia cases in China. This virus is spread by coughing or sneezing and can infect other persons by on contacting mucous membranes. SARS-Cov-2 most frequent serious manifestation is pneumonia. Chest computed tomography in COVID-19 patients usually shows ground-glass opacities that may be accompanied by consolidation lesions. Early diagnosis of the disease and rapid isolation of the patient is of great importance. So far, confirmation of COVID-19 infection is made by RT-PCR of nasopharyngeal or respiratory specimens. Recent research reported that the sensitivity of computed tomography in diagnosing COVID-19 is 98% while RT-PCR sensitivity is 71%. Herein, we compare the sensitivity of both chest CT and RT-PCR in diagnosing COVID-19 at initial patient presentation through a meta-analysis study.

**Main body:**

Using MEDLINE database a systematic literature search was conducted to identify relevant published studies within from November 2019 to April 2020. Only articles with full text were examined to determine eligibility and extract data by two reviewers. It was decided to include studies mentioning sensitivity of chest CT scan and sensitivity of RT-PCR and both done at the same time.

**Results:**

Potentially relevant 15,300 studies were identified in our search in MEDLINE whose titles were quickly reviewed. Potentially eligible studies missing any of the forementioned inclusion criteria were excluded. This process left 7 eligible articles that fulfilled the inclusion criteria and were thus included in the meta-analysis and used for further analyses.

**Conclusion:**

The meta-analysis study showed that chest CT may be beneficial in early detection of cases of COVID-19. Imaging, in adjunct to clinical and laboratory findings, should be used for monitoring of disease course, until further evidence is available.

## Background

A new coronavirus was known, at the end of 2019, to cause a collection of pneumonia cases in Wuhan, China. As of the beginning of 2020, the virus spread rapidly resulting in a worldwide pandemic. The virus causing COVID-19 was named severe acute respiratory syndrome coronavirus 2 (SARS-CoV-2) [1]. The incubation period of SARS-CoV-2 infection is around 14 days; however, most of the cases show symptoms after 4 to 5 days of exposure [2–4]. Pneumonia is the most common extreme presentation of SARS-CoV-2 infection, manifesting by fever, dry cough, difficulty of breathing, and ground-glass infiltrates in radiological images [3, 5–7]. No unique clinical features can yet be used to differentiate COVID-19 from other pneumonic viral respiratory infections. CT imaging in COVID-19 patients commonly shows ground-glass opacities.

Sometimes accompanied by areas of consolidation, findings are consistent with viral pneumonia [8, 9]. A recent case series reported that chest CT findings are more frequently to occur bilateral, have a peripheral distribution, and comprise mainly the lower lobes. Less common findings in COVID-19 patients include pleural thickening and pleural effusion [1].

In the absence of specific anti-viral drugs or vaccines for SARS-CoV-2, early detection of the disease and the instant isolation of an infected patient are crucial.

The National Health Commission of China states that the diagnosis of SARS-CoV-2 infection must be established by viral nucleic acid detection either by reverse-transcription polymerase chain reaction (RT-PCR) or by sequencing of either respiratory or blood samples [10]. The total positive rate of PCR for throat swab samples is reported to be about 30 to 60% at the original presentation and this is mainly caused by confines regarding sample collection, sample transport, and kit performance [11].

Due to the lack of presence of RT-PCR in the current public health emergency, many COVID-19 patients are not identified quickly and are not receiving the appropriate treatment. In addition, as they do not know that they are infected and given the highly transmissible nature of the virus, they do bear a chance of infecting others. Chest CT may be a more accurate, available, realistic, and fast method for diagnosing and assessing suspected COVID-19 patients compared to RT-PCR. A recent study reported the sensitivity of CT to be 98% in diagnosing COVID-19 infection compared to RT-PCR which showed a sensitivity of 71% [11].

In this study, we aimed to compare the sensitivity of chest CT to the sensitivity of RT-PCR at the initial patient presentation through a meta-analysis study.

## Methods

### Search for relevant studies

We used the MEDLINE database (www.pubmed.com) to perform a systematic search of literature to find relevant studies that were published within the past 6 months (from November 2019 to April 2020). All appropriate articles were accessed in full text in order to determine the eligibility and extract the data by two reviewers.

We also scanned the references of the retrieved articles to find further studies that could have been missed in our initial search, the online searches were expanded. We agreed to only include studies that are reported or translated in English and that address chest CT scan sensitivity and RT-PCR sensitivity and both performed simultaneously (time of initial presentation). We excluded articles that missed one or more of the forementioned inclusion criteria, duplicated studies, or those outdated by newer ones and studies with provided data that cannot be extracted.

### Study selection and data abstraction

From each article, the subsequent information was abstracted: type of the study (meta-analysis or randomized control trials, prospective, retrospective, and systematic review), sensitivity of chest CT scan, and sensitivity of RT-PCR.

### Statistical methods

Statistical analysis was completed using the jamovi version 1.1 computer software (the jamovi project. jamovi version 1.1, 2019. Recovered from https://www.jamovi.org).

Data were acceptable for conducting meta-analysis for the CT scan or RT-PCR sensitivity. Other accuracy indices could not be assessed.

Studies included in meta-analysis were tested for heterogeneity of the estimates by means of the Cochran *Q* chi-squared test and *I*-squared statistic.

Publication bias was judged by analyzing funnel plots of the estimated effect size versus its standard error. To examine the asymmetry of funnel plots, the Begg-Mazumdar rank correlation and the Egger regression tests were used.

Effect sizes were pooled using random-effects maximum likelihood model. Pooled sensitivity is reported with 95% confidence limits and prediction interval.

## Results

### Study identification and eligibility

Our search recognized 15,300 potentially relevant studies in MEDLINE whose titles were quickly reviewed. Records after duplicates removed were 10,100 articles. There were 200 studies that were potentially eligible from them.

Out of the 200 studies, 150 were excluded as they lack one or more of the forementioned inclusion criteria or as they were outdated by other more recent ones. So, 50 studies were eligible for possible inclusion and were accessed in the full-text form. After going through the full length of the articles, 43 studies were excluded as some of them were essay studies while others did not mention the sensitivity of one or both researched items. This process left 7 original articles that fulfilled the inclusion criteria and were thus included in the meta-analysis and used for further analyses, PRISMA diagram [1].

### Analysis of included articles

**Fig. 1:**
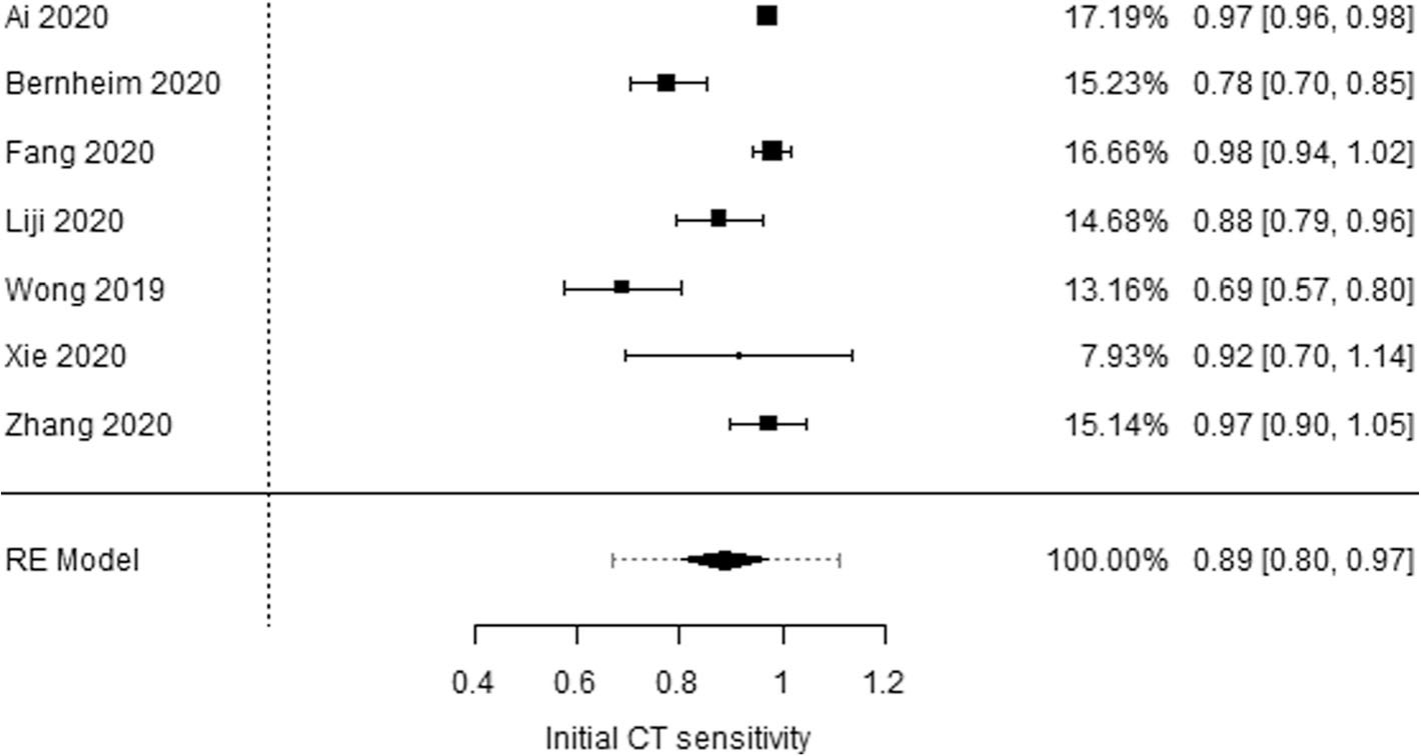
Forest plot for sensitivity of initial CT scan. There is considerable heterogeneity across studies (Cochran *Q* test *P* value < 0.001, *I*-squared = 94.2%). Pooled sensitivity = 89% (95% CI = 80 to 97%). Prediction interval is presented as a dotted extension up and down the 95% confidence limits of pooled effect size [11–17]

**Fig. 2:**
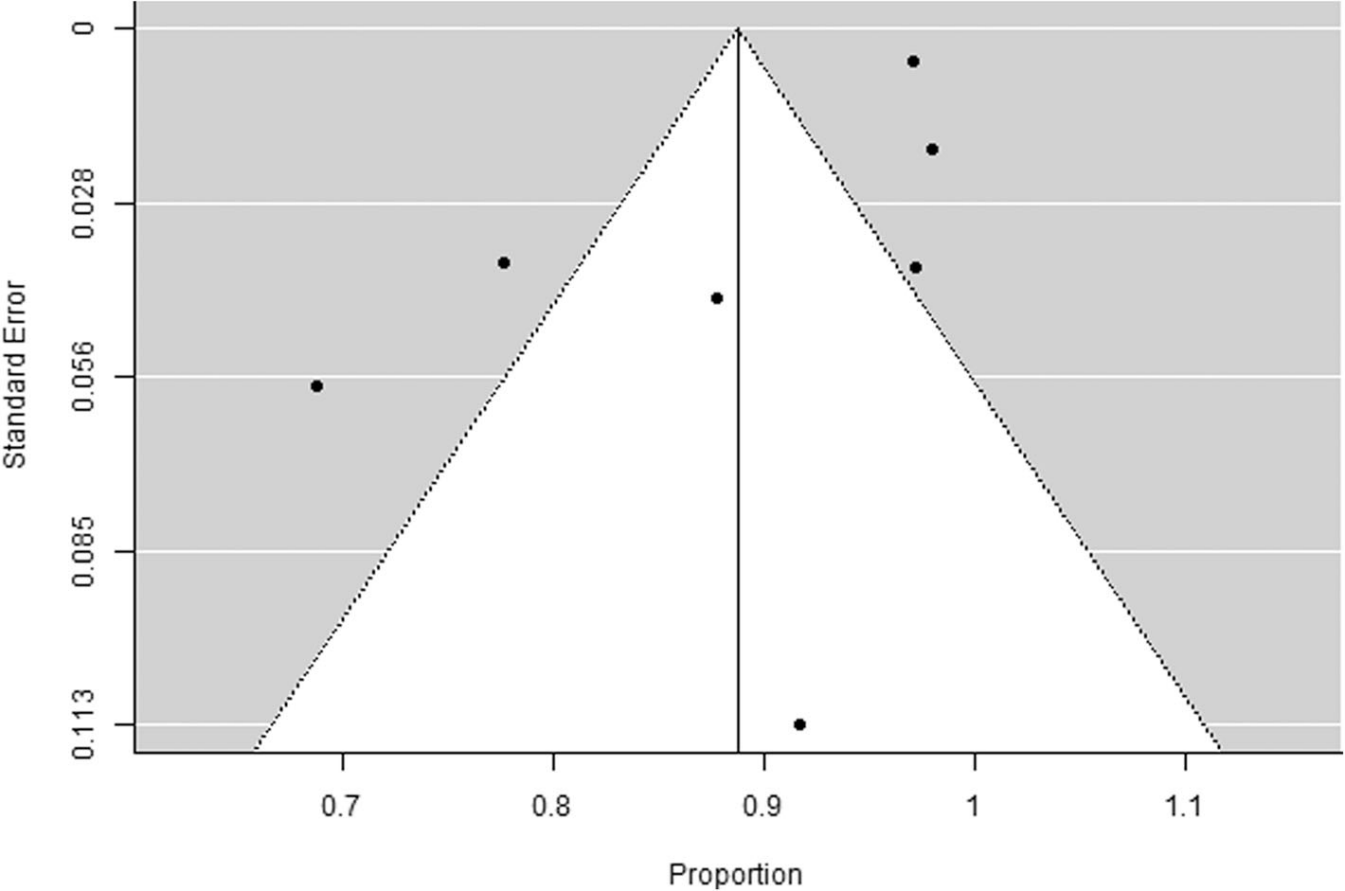
Funnel plot for sensitivity of the initial CT scan. There is no evidence of publication bias (Rosenthal fail-safe number = 37,526, rank correlation test for funnel plot asymmetry *P* value = 0.239, regression test for funnel plot asymmetry *P* value = 0.300)

**Fig. 3:**
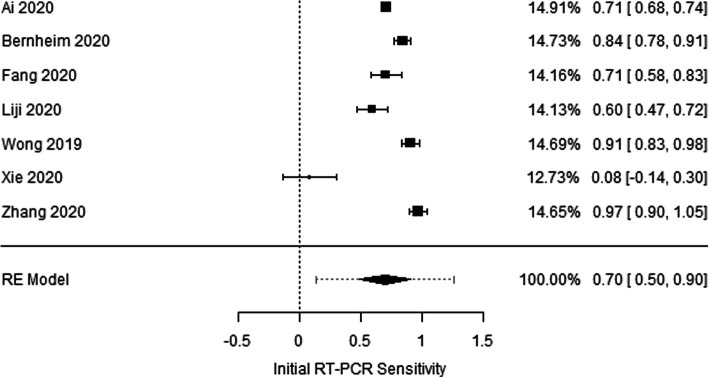
Forest plot for sensitivity of initial RT-PCR. There is considerable heterogeneity across studies (Cochran *Q* test *P* value < 0.001, *I*-squared = 98.2%). Pooled sensitivity = 70% (95% CI = 50 to 90%). Prediction interval is presented as a dotted extension up and down the 95% confidence limits of pooled effect size [11–17]

**Fig. 4:**
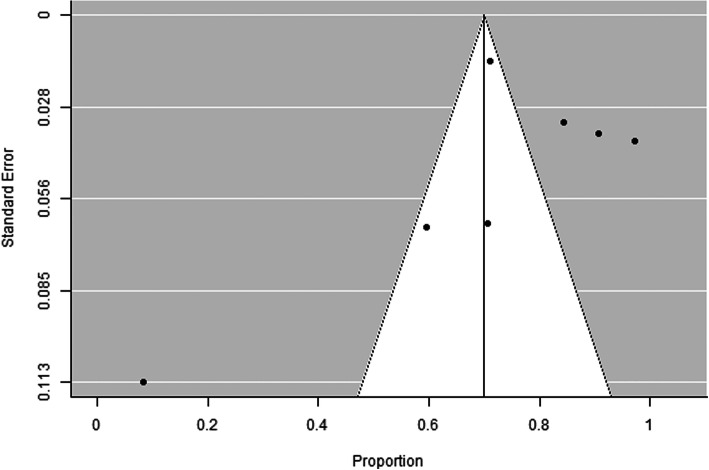
Funnel plot for sensitivity of the initial CT scan. There is possibility of publication bias (Rosenthal fail-safe number = 7902, rank correlation test for funnel plot asymmetry *P* value = 0.562, regression test for funnel plot asymmetry *P* value = 0.002)

Among the 7 included articles, there were no randomized control studies. Only retrospective studies were found and used for additional analysis, Figs. 1, 2, 3 and 4, and.

PRISMA diagram [1]

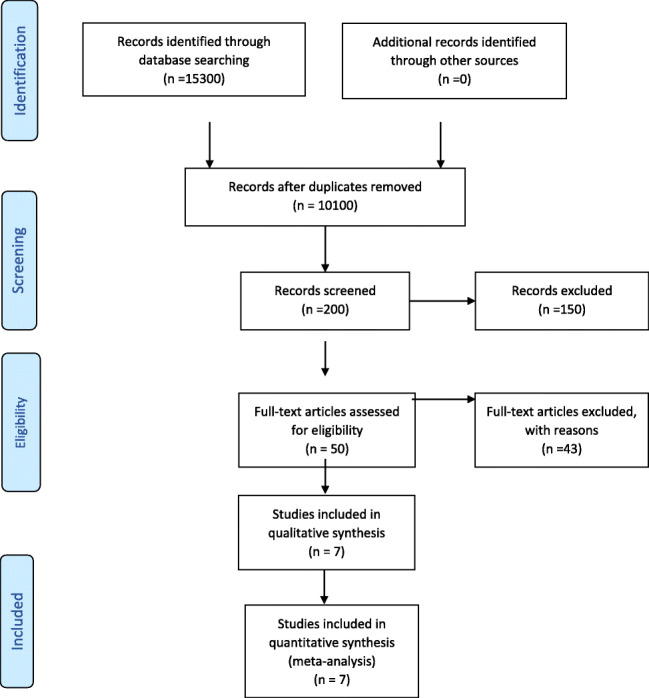



## Discussion

The absence of specific anti-viral drugs or an anti-SARS-CoV-2 vaccine necessitates the early diagnosis of COVID-19 to allow efficient disease control and treatment. Chest CT imaging could be a fast method, compared to RT-PCR, to early detect and assess COVID-19, particularly in the epidemic zone [11]. Clinicians are assessing more and more suspected patients, and so are radiologists who are correspondingly interpreting more and more chest CTs of patients suspected to have COVID-19. Clinical practice has shown that chest CT is an important component that aids in the diagnosis of patients with suspected COVID-19 infection. In fact, given the inadequacy of RT-PCR kits in some health facilities and the likelihood of occurrence of a false negative RT-PCR result for SARS-CoV-2, the National Health Commission of the People’s Republic of China is encouraging diagnosis based on clinical findings in addition to chest CT findings alone [18].

In this meta-analysis, we aimed to investigate the sensitivity of chest CT scan versus the sensitivity of RT-PCR in diagnosing COVID-19 infection at initial presentation.

Seven articles were eligible to this study. Forest plot test for sensitivity of initial CT scan indicates considerable heterogeneity across studies (Cochran *Q* test *P* value < 0.001, *I*-squared = 94.2%), and the pooled sensitivity was 89% (95% CI = 80 to 97%). Also funnel plot for sensitivity of initial CT scan shows no evidence of publication bias (Rosenthal fail-safe number = 37,526, rank correlation test for funnel plot asymmetry *P* value = 0.239, regression test for funnel plot asymmetry *P* value = 0.300) (Figs. 1 and 2). On the other hand, the forest plot test for sensitivity of initial RT-PCR shows considerable heterogeneity across studies (Cochran *Q* test *P* value < 0.001, *I*-squared = 98.2%) and pooled sensitivity = 70% (95% CI = 50 to 90%). Funnel plot for sensitivity of initial RT-PCR scan declares the possibility of publication bias (Rosenthal fail-safe number = 7902, rank correlation test for funnel plot asymmetry *P* value = 0.562, regression test for funnel plot asymmetry *P* value = 0.002) (Figs. 3 and 4).

Review and analysis of the data collected from the included 7 articles revealed that RT-PCR testing for viral nucleic acid plays a vital role in defining hospitalization and isolation for individual patients. However, it is less sensitive than chest CT (89% versus 70% respectively). Additionally, many external aspects can affect the result of RT-PCR test including sampling technique, specimen type (upper or lower respiratory tract), time of sampling (different period of disease development) [19], in addition to the performance of the used detection kits. Hence, RT-PCR test results must be interpreted with care as such.

Chest CT is a non-invasive, conventional imaging technique with high precision and speed. Based on recent published literature, nearly all patients with COVID-19 have distinctive CT features during the disease process [8, 11, 12, 20, 21], e.g., variable degrees of ground-glass opacities that may be accompanied by crazy-paving sign, bilateral multifocal organizing pneumonia, and architectural alteration in a peripheral distribution. Liji Thomas stated that CT picked up nearly all cases that were detected using RT-PCR, and 75% of the cases that were initially missed by serological test. The authors reported that chest CT had a positive predictive value of 65% and a negative predictive value of 83%. He also reported that in spite of a relatively high number of false positive, the priority in such an emergent situation is to identify the biggest number of cases as hastily as possible rather than absolute accuracy of diagnosis [14].

In a study performed by Ai, T et al., 60% (34/57) of the cases had characteristic CT findings that are consistent with COVID-19 either prior to or parallel to the positive RT-PCR results. Also, nearly all patients (56/57) had initial chest CT findings before or within 6 days of the initial positive RT-PCR results showing that chest CT might be very valuable in the early detection of suspected cases [11].

It was proven that the standard diagnostic method being used is real-time polymerase chain reaction (RT-PCR) for spotting viral nucleotides from specimens collected by oropharyngeal swab, nasopharyngeal swab, bronchoalveolar lavage, or tracheal aspirate [19]. Nonetheless, new reports have shown that RT-PCR has a sensitivity as small as 60-71% for detecting COVID-19 [11, 13, 20]. RT-PCR test results for COVID-19 may be false negative either due to laboratory errors or due to insufficient presence of viral material in the clinical sample [13, 16]. Moreover, the current laboratory test still remains time consuming; moreover, a shortage in kit supply might not meet the growing needs of the infected population.

Such false negatives prolong quarantine attempts, require repeated testing and have the potential to overwhelm the current test kits and associated infrastructure supply [20]. By comparison, chest CT has demonstrated a sensitivity of about 56-98% in the identification of COVID-19 at preliminary presentation, and might be helpful in the correction of false negatives obtained from RT-PCR test during the early disease phase [13, 20].

Fang et al. stated that the sensitivity of chest CT in their analysis was superior to that of RT-PCR (98% versus 71%, respectively, *P* < .001). The authors attributed the reason for the low performance of RT-PCR to (1) the novelty of nucleic acid testing that still might need enhancement; (2) disparity in detection level from different suppliers; (3) patients who might have viral load; or (4) incorrect clinical sampling. They concluded that their results do support the use of chest CT in screening of COVD-19 patients with either clinical and/or epidemiologic features indicative of COVID-19 infection especially when RT-PCR test is negative [13].

Recently, their result was supported by Xie who reported that 5/167 (3%) patients had initially negative RT-PCR for COVID-19 in spite of chest CT findings characteristic of viral pneumonia at earlier presentation [16].

Shi and colleagues studied patients confirmed to have SARS-CoV-2 infection and reported abnormal chest CT findings even in asymptomatic patients. Obviously, their findings are important for the early clinical management of COVID-19 pneumonia patients. From an epidemiological point of view, however, CT analysis for early detection of SARS-CoV-2 infection needs to be done with caution [22].

On the other hand, Bernheim et al. stated that 56% of patients imaged early, (0-2) days from onset of symptoms, had normal CT appearance as compared to 9% of intermediate patients (3-5) days and 4% of late patients. So, early after symptom onset, chest CT has imperfect sensitivity and negative predictive value and is so unlikely a reliable standalone means to rule out COVID-19 infection [12]. Also, Chung et al. reported negative imaging in known infected patients COVID-19 at initial presentation and CT appearance of COVID-19 that shares similar findings with other diseases that cause viral pneumonia (3/21 patients) [20].

Another study reported a clashing finding that 7/19 (37%) asymptomatic cases, that had positive RT-PCR results, had no CT changes [23]. This data shows the limited value of CT screening in early COVID-19 diagnosis. Hence, we have sufficient reasons to question if CT is a suitable means for screening of asymptomatic infections. Additionally, studies have also shown that the 2ry attack rate among close contacts is 9.6% (95% CI 7.9–11.8) [3], and that asymptomatic patients account for only around 1-2% of total SARS-CoV-2 infections [4].

The American College of Radiology (ACR) opposes the results of meta-analysis study and said that “CT chest should not be used to screen for or as a first-line for Covid-19 diagnostic test.”

The ACR believes that the following factors considerations about the use of imaging for suspected or known COVID-19 infection:

- The Centers for Disease Control (CDC) currently does not recommend plain chest X-ray or CT to diagnose SARS-CoV-2 infection. Nucleic acid viral testing remains the only specific method of diagnosis. Even if COVID-19 radiologic findings are suggestive, confirmation with the viral test is required.
- Generally, chest X-ray or chest CT findings of COVID-19 are not specific and do overlap with findings seen in other infections, including influenza, H1N1, SARS, and MERS. The presence of a flu season with a much higher prevalence of influenza in the US than COVID-19, further limits the specificity of CT [24]*.*

The Royal College of Radiologists announced that “there is no existing role for CT in the diagnostic assessment of patients with suspected infection with coronavirus in the UK.” This was in March 12, 2020; however, in March 27, they said, chest CT assessment for the presence of likely COVID-19 infection might be beneficial in stratifying risk in acutely presenting patients. In the absence of quick access to COVID testing, this is suitable if it will affect the patient management. However, a negative CT scan result would not exclude COVID-19 infection. As with all other advice now, this may change [25].

The Royal Australian and New Zealand College of Radiology advised that “CT should not be used for routine screening for Covid-19 disease.” [26].

The Canadian Association of Radiologists stated in March 26, 2020, that a normal chest CT scan cannot exclude COVID-19 particularly for patients with recent onset of symptoms. According to the Public Health Agency of Canada (PHAC) and the World Health Organization (WHO), the final diagnosis of COVID-19 infection should be confirmed by a positive RT-PCR test. This is standard of reference. The Canadian Society of Thoracic Radiology (CSTR)/Canadian Association of Radiologists (CAR) recommends against the use of routine chest CT for screening, diagnosis, and surveillance of COVID-19 infection. The CSTR/CAR recommends chest CT in patients with confirmed COVID-19 infection who may have acquired complications such as a lung abscess or empyema [27].

To date, most radiologic data has been coming from China. Some studies suggest that chest CT in the setting of a negative PCR test may be positive. Other studies deny the benefit of chest CT in the early diagnosis of COVID-19. All over the world, knowledge is still rapidly evolving and not all available information are published and or publicly shown. So, the possibility of using chest CT in early diagnosis of COVID-19 still needs more supportive data and also the use of chest CT in screening high-risk groups should be weighed against risks and benefits to reduce radiation dose. Clear criteria for the use of CT in diagnosis of COVID-19 should be established based on current available data. One criteria would be having symptoms or signs suggestive of infection or having contact with a diagnosed patient and have a positive RT-PCR test; another would be to treat or determine the course of the disease.

## Conclusion

The meta-analysis study revealed that chest CT may be beneficial in early detection of cases of COVID-19. Further studies from different centers all over the world should be waited. Until that time imaging should be used as an adjunct to clinical and laboratory parameters in monitoring of disease course, until additional evidence is available.

## Data Availability

Available upon request

## Abbreviations

RT-PCR: Reverse transcriptase-polymerase chain reaction
CT: Computerized tomography
COVID-19: Coronavirus disease of 2019
CXR: Chest X-ray
SARS-CoV-2: Severe acute respiratory syndrome coronavirus 2
PRISMA: Preferred Reporting Items for Systematic Reviews and Meta-Analyses
